# Antibiotic-Loaded Calcium Sulphate Beads for Treatment of Acute Periprosthetic Joint Infection in Total Knee Arthroplasty: Results Based on Risk Stratification

**DOI:** 10.3390/jcm14051531

**Published:** 2025-02-25

**Authors:** Edward J. McPherson, Madhav Chowdhry, Matthew V. Dipane, Benedikt Marahrens, Diego Dela Pena, Alexandra I. Stavrakis

**Affiliations:** 1 Department of Orthopaedic Surgery, David Geffen School of Medicine, University of California (UCLA), Los Angeles, CA 90404, USA; emcpherson@mednet.ucla.edu (E.J.M.); mdipane@mednet.ucla.edu (M.V.D.); dpena@mednet.ucla.edu (D.D.P.); astavrakis@mednet.ucla.edu (A.I.S.); 2 Department of Continuing Education, University of Oxford, Oxford OX1 2JA, UK; 3 Department of Internal Medicine, Brandenburg Medical School, Neuruppin, 14770 Brandenburg, Germany; benedikt.marahrens@uk-brandenburg.de

**Keywords:** total knee arthroplasty, TKA, periprosthetic joint infection, PJI, acute infection, modular exchange, component retention, DECRA, DAIR, antibiotic-loaded calcium sulphate beads

## Abstract

**Background:** A post-operative or late acute periprosthetic joint infection (PJI) after Total Knee Arthroplasty (TKA) requires a protocol of aggressive joint Debridement, modular implant Exchange, Component Retention, and post-operative Antimicrobial therapy (DECRA). Recently, the novel addition of intra-articular Antimicrobial Loaded Calcium Sulphate (AL-CaSO_4_) beads during DECRA has been utilized to improve microbial eradication. This study reviews a consecutive series of DECRA TKA procedures with AL-CaSO_4_ beads with a standardized antimicrobial regimen. We hypothesize AL-CaSO_4_ beads will not improve infection-free implant survival in compromised hosts and limbs. **Methods:** This consecutive series included DECRA procedures for acute post-operative or late hematogenous PJI-TKA (primary and revision) detected within 4 weeks. One gram of vancomycin powder and 240 mg of liquid tobramycin were added to 10 cc of CaSO_4_ powder to create 3.0 and 4.8 mm beads delivered into the joint at closure. All patients were risk stratified according to McPherson Staging and followed for a minimum of 1 year. **Results:** Forty-two patients were studied. The infection-free success rate of DECRA with AL-CaSO_4_ was 62% (26/42) at 1 year. Average bead volume per case was 18.6 cc (range = 10–40 cc). McPherson Host stage and Limb Score were found to be significantly correlated with the success of the DECRA (*p* < 0.05). The success rate was highest in A-hosts (87.5%), declining to 50% in B-hosts, and 25% in C-hosts. Similarly, the success rate was highest for patients with Limb score 1 (100%), declining to 58.6% with Limb score 2, and 20% with Limb score 3. Importantly, a previous episode of infection in the affected joint was associated with significantly increased failure (*p* = 0.000025). **Conclusions:** This study reports an overall higher infection-free success rate of DECRA using AL-CaSO_4_ beads compared to the current literature. Antibiotic beads provide an advantage in selected groups that include A or B hosts and Limb scores of 1 or 2. In C-hosts, where the immune system is weak, or Limb score 3, where the wound is compromised and leaks, antibiotic beads do not improve success. Importantly, DECRAs should not be considered curative with a prior history of joint infection. In these difficult circumstances, one should consider an exchange protocol.

## 1. Introduction

Periprosthetic Joint Infection (PJI) following Total Knee Arthroplasty (TKA) is a challenging complication having immense impact upon patients and healthcare systems. For the patient, this could be the beginning of a long fight against resistant microbes that may culminate with limb ablation. For healthcare systems, the financial burden is immense, with compounding costs for treatment. It is estimated that the direct annual hospital costs for treatment of hip and knee PJI is projected to exceed 1.85 billion USD by 2030 in the United States alone [1,2].

PJI is classified into two distinct states, acute or chronic. Chronic PJI is characterized by a robust bacterial adherence with a mature biofilm, conferring substantially increased resistance to antimicrobial therapy. Effective treatment requires an aggressive implant exchange protocol [3,4,5,6,7]. In contrast, an acute PJI is characterized by an evolving state of microbial colonization. It is broadly accepted that a mature biofilm is not established, and the infected joint exists in a “Biofilm Transitional State” (BTS). Thus, in an acute PJI, most agree that implants can be salvaged if treated early (≤4 weeks) [8]. The treatment most advocated is radical joint Debridement, Exchange of all modular parts (bearings, heads, screws, etc.), with Component Retention, and post-operative Antimicrobial therapy (DECRA). We distinguish DECRA specifically from DAIR (Debridement, Antibiotics & Implant Retention), as a DAIR may not include modular exchange, especially in countries where there are financial constraints. The success of a DECRA procedure depends on multiple factors. These include: (1) early surgical intervention before mature biofilm formation; (2) the quality and extent of surgical debridement of peri-articular tissues; (3) ability of the host to mount an effective immune response; and (4) effective post-operative antimicrobial treatment. Not all DECRA procedures are successful. One problem is surgical debridement. Surgeons must respect the surrounding periprosthetic neurovascular structures and preserve ligamentous/capsular structures, which may allow microbes to survive in surgically unreachable areas [9].

Local peri-articular antimicrobial delivery with dissolvable antibiotic loaded CaSO_4_ (AL-CaSO_4_) beads using a combined antimicrobial regimen (vancomycin with an aminoglycoside being the most common) has been used as adjunctive treatment in acute PJI [10,11]. The premise for local delivery with beads is threefold. First, it allows high antimicrobial concentrations within a joint [10,11]. Second, it can allow antimicrobial diffusion into surgically inaccessible areas. Lastly, AL-CaSO_4_ beads allow for prolonged elution as compared to applying an antibiotic powder into the joint which rapidly dissipates. An in-vitro large joint modeling study documents antimicrobial levels with AL-CaSO_4_ beads far above biofilm killing levels, with antibiotic levels within the test fluid extending up to 6 weeks. Augmenting the DECRA procedure with this strategy may improve infection-free implant survival in acute PJI. However, adjunctive bead application may not be protective in compromised host and limb conditions [8,12].

This study reviews a consecutive series of DECRA procedures for acute, post-operative, and late hematogenous PJI treated with adjuvant intra-articular AL-CaSO_4_ beads. We stratified our patients according to Host Stage, Limb score, and type of index surgery (primary vs. revision) [8,12]. We hypothesize that antibiotic-loaded beads will not improve infection-free implant survival in compromised hosts and limbs.

## 2. Materials and Methods

A retrospective review was done for a consecutive series of DECRA procedures performed at a tertiary care center from January 2018 to January 2020. All patients with acute PJI after TKA (both primary and revision) undergoing DECRA procedure were included for study. PJI was defined according to the 2013 ICM definition of PJI [13]. Acute PJI was defined as an infection within 4 weeks of index surgery or late-onset acute hematogenous infection with a symptom duration ≤ 4 weeks. Any patient with symptom duration > 4 weeks or with radiographic signs of bone erosion was excluded. Each patient was categorized according to McPherson Staging system for assessing the host and limb health [8,12]. Institutional Review Board (IRB) approval (IRB#23-001087) was received prior to the review and informed consent was obtained from all participants.

*DECRA Procedure:*

The knee was approached with an extended paramedial arthrotomy (typically 15–20 cm). A minimum of 4 cultures were taken intra-operatively including fluid, synovium, and periprosthetic bone tissue at the implant edges. The knee was flexed with the patella everted for complete exposure. A total synovectomy was performed that started at the most superior aspect of the suprapatellar pouch to the proximal tibia. All modular parts were removed and subsequently exchanged with new ones. With the bearing removed, a posterior synovectomy was performed. Implant surfaces were physically scrubbed and the entire joint was pulse-lavaged with 9 L of sterile Normal Saline (NS) solution.

*Antibiotic-Loaded CaSO_4_ Bead Preparation:*

**Figure 1:**
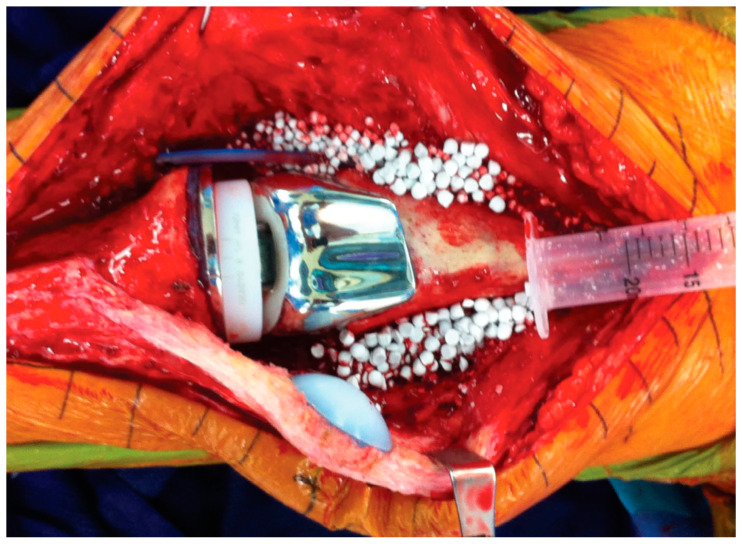
Figure showing uniform distribution of dissolvable AL-CaSO_4_ beads around the knee joint after completion of the DECRA procedure.

The CaSO_4_ product used for this study was Stimulan^®^ (Biocomposites, Keele, United Kingdom). We used the 10 cc bead kits that consisted of CaSO_4_ powder mixed with 1 g vancomycin powder and 240 mg tobramycin liquid, as the antimicrobial agents. The paste was spread into sterile silicon mats allowing for the fabrication of 3-, 4.8-, or 6-mm beads. For this study, we used the 3.0- and 4.8-mm beads. At the completion of the DECRA procedure, the dissolvable AL-CaSO_4_ beads were uniformly distributed around the knee joint (Figure 1). The beads were strategically placed to avoid interposition into the prosthetic articulation.

All patients received intra-venous (i.v.) post-operative antibiotics according to the culture-sensitivity of the pre-surgical aspirate and/or intra-operative cultures. If all cultures were negative, then patients were treated empirically with i.v. vancomycin. IV antibiotics were transitioned to oral antibiotics at 4 weeks. The total duration of antimicrobial treatment was 6 weeks. The success of the procedure was defined as being clinically infection-free, with retention of original components for a duration of 1 year from the DECRA procedure. This was assessed with serum biomarkers measuring erythrocyte sedimentation rate and c-reactive protein, and a knee aspiration with synovial fluid culture. Treatment failure was defined as infection requiring implant removal or limb amputation. Chronic antimicrobial suppression therapy was not used in the study.

*Statistical analysis:*

Statistical analysis was performed using SPSS version 28.0 (IBM SPSS Statistics, Chicago, IL, USA). Categorical variables were compared using a chi-square test. Multivariate logistic regression was performed to correlate host-related, limb-related, and time-related factors with the success of surgery. A *p*-value < 0.05 was considered statistically significant.

## 3. Results

A total of 42 patients met the inclusion criteria for the study. There were 27 males and 15 females in this study with the mean age of 65.3 years (range 56–82). The overall success rate of DECRA was 62% (26/42). The average duration of symptoms in this group was 7.4 days (range 1–24 days). The average quantity of bead volume used in each case was 18.6 cc (range 10–40 cc). Of the 26 successfully treated patients in this group, only 15 patients had microbial growth on intra-operative culture. Three patients tested positive for polymicrobial infection. The most common organisms identified were Staphylococcus epidermidis and Staphylococcus aureus.

Sixteen out of 42 patients (38%) failed the DECRA procedure. The average duration of symptoms before presentation in this group was 7.3 days (range 2–21). The average quantity of bead volume used in this group was 22.8 cc (range 15–30 cc). Intra-operative cultures were positive in 12 out of 16 patients in this group, two of whom had a polymicrobial growth. The most common organism in this group was Staphylococcus aureus.

Multivariate logistic regression was performed to correlate host-related, limb-related, and time-related factors with the success of surgery (Table 1). McPherson Host stage and Limb Score were found to be significantly correlated with the success of the DECRA procedure (*p* < 0.05). More importantly, a previous episode of infection in the affected joint was associated with significantly increased failure (*p* = 0.000025).

Stratifying patients to Host Stage, the success rate of the DECRA procedure was highest in A-hosts 87.5% (14/16), declining to 50% (11/22) in B-hosts, and 25% (1/4) in C-hosts. Similarly, the success rate was highest for patients with Limb score 1 100% (8/8), declining to 58.6% (17/29) with Limb score 2, and 20% (1/5) with Limb score 3. In patients without a prior history of joint infection, the success rate was 95% (19/20), which declined to 32% (7/22) in patients with a prior history of joint infection (Table 2).

## 4. Discussion

Musculoskeletal infections (MSI) are becoming more problematic to treat. The reasons for this include: the ongoing adaptive resistance mechanisms selected out by the persistent use of systemic broad-spectrum antimicrobial agents, the increasing use of immune-modulating agents that inactivate parts of the host’s adaptive and innate immunity, and the ever-progressing use of sophisticated metallic/ceramic implants for musculoskeletal reconstruction, which can harbor micro-organisms within and around implants [9,14,15,16]. In the last two decades, the success rates of treating Periprosthetic Joint Infection (PJI) has not significantly improved despite the aggressive use of stronger parenteral antibiotics [17,18]. In fact, we believe that the use of parenteral antibiotics has a deleterious effect on the host’s immune response, as these antibiotics disrupt the microbial biome within the gut and upon the surface of the human body [19,20,21]. The communication between microbial biome and humans is complex and the disruption of this equilibrium shifts the advantage towards microbial survival [22,23,24,25]. We believe that treatment requires innovation. In the specific area of PJI, one tactic used involved local antimicrobial delivery via local insertion of dissolvable AL-CaSO_4_ in a small bead format (3–8 mm) following debridement. The beads were mixed by the surgeon at the time of surgery, with point of care delivery to the infection site at surgical closure [11,26,27]. Compared to delivery via the parenteral route, local delivery has purported to increase intra-articular antimicrobial levels, allowing for more effective diffusion to the entire infection zone [10,28,29]. Furthermore, local delivery lessens systemic antimicrobial exposure preserving host-microbial equilibrium.

Historically, DECRA procedures for acute PJI have shown poor infection-free implant survivorship. Despite established debridement protocols at major centers, the combined survivorship of DECRA procedures have ranged from 32–48% [30,31]. We believe this relates to the inadequate diffusion of parenteral antimicrobial agents into the extended zone of infection (ZOI). At some centers, these poor results have influenced treatment protocols, shifting the treatment of acute PJI towards implant exchange protocols in lieu of DECRA, when PJI has been present for more than 2 weeks. However, exchange protocols are difficult and are associated with morbidity and long-term functional limitations [32,33,34,35,36]. Instead, improving the DECRA technique by employing adjuvant methods to improve microbial kill has become an area of increasing focus. Recent additions to DECRA include the use of more powerful multi-modal wound lavage agents and the addition of AL-CaSO_4_ beads placed into the joint at the time of closure [32,37,38,39,40,41,42].

To this point, we used periarticular dissolvable AL-CaSO_4_ beads with a set antibiotic formula of vancomycin and tobramycin to supplement DECRA-assessing infection-free implant survival. We chose this combination for two reasons. First, vancomycin with an aminoglycoside provides synergy, as the dual mechanisms of cell wall disruption and inhibition of 30S ribosomal translation, increases the effective kill of many microbial species [10,11]. Second, this combination provides the best broad-spectrum coverage against organisms typically identified in PJI [43,44,45,46]. We note that many PJIs are culture-negative for reasons including the concurrent use of antibiotics, fastidious organisms that do not survive transport to the lab, and poor sampling technique. Moreover, next generation DNA sequencing of infection samples has identified more microbial species than what is detected in culture [37]. Thus, our vancomycin-tobramycin combination provides the best microbial coverage when microbial identification is not known. Because they are placed locally, AL-CaSO_4_ beads have the advantage of eluting high concentrations of antimicrobial agents locally with a lower risk of systemic toxicity. Moreover, by diffusion, local antimicrobial levels are high enough to reach out to surgically inaccessible areas of infected tissue, and into tissues with compromised vascularity, providing a wider periprosthetic microbial kill zone [29,38].

However, the use of AL CaSO_4_ beads does incur risk. We caution that high volume (>20 cc) use of AL-CaSO_4_ beads can cause increased wound drainage related to increased intra-articular acidity, and joint effusion related to inflammatory reactions with bead dissolution [11,47]. Thus, patients with compromised limb conditions with poor healing, may be unable to tolerate antimicrobial bead treatment. Further, we note the final termination of a PJI rests with the host immune system. No matter how effective the debridement and efficiency of local antimicrobial delivery is, the host must have an immune system competent enough to thwart any microbial regrowth and re-establishment of a PJI.

Our overall success of DECRA was 62%, which is distinctly higher compared to the noted published literature. We attribute our improved success to the addition of AL-CaSO_4_ beads to the DECRA. More importantly, in the stratified analysis of our data, we found Host and Limb scores to be important factors in the success of DECRA. In addition, we note an increased failure rate in those patients with a prior history of infection in the affected joint (*p* = 0.000025). This suggests that a DECRA procedure is unlikely to cure infection in the face of a prior history of joint infection. With a prior infection, microbes can persist within the bony osteo-canalicular network in areas not accessible to antimicrobial diffusion and immune cell ingress [48,49,50,51]. Based on the above findings, we believe in selectively using DECRA with dissolvable AL-CaSO_4_ beads when there is a good chance for successful microbial eradication. Specifically, we advocate DECRA with AL-CaSO_4_ beads as primary treatment for acute PJI in A- and B-Hosts, Limb score 1 and 2. In C-hosts, the immune system is too weak to clear microbial reserves. In Limb score 3, the surrounding soft tissue envelope is weak, allowing microbes to ingress. In these conditions, high local antibiotic concentrations will not be curative. In fact, high concentrations will only select out resistant organisms. We strongly recommend a prosthetic exchange protocol (or when circumstances warrant, limb ablation) for C-hosts, Limb score 3 and those with a prior history of infection of the affected joint. We acknowledge that there are extenuated circumstances where an exchange protocol would be insurmountable for ill patients and a DECRA procedure would be warranted. In these conditions, the DECRA would be palliative and require oral suppressive antimicrobial therapy.

This study has several limitations. First, this study is of small sample size, so our recommendations should be taken with caution until further studies corroborate our observations. However, this report is still one of the larger single center studies providing scrutiny of the DECRA technique. Second, treatment was performed at a single tertiary care center led by a high-volume surgeon with extensive experience in treating PJI. This factor may have contributed to the comparatively higher success rate of DECRA with AL-CaSO_4_ beads. However, we note that at all large centers treating PJI, debridement techniques are comparable. Further, the addition of AL-CaSO_4_ beads to DECRA was in response to our center’s prior failures of DECRA. This study did not incorporate a matched control group, as with our database we were unable to reach retrospectively far enough for control matching. Lastly, a minimum duration of 1 year was selected for the follow-up assessment. One year follow-up may limit the capture of late recurrences, but our experience suggests that in the treatment of acute PJI, most of all recurrences are identified within the one-year follow-up interval. Definitive assessment of DECRA employing AL-CaSO_4_ beads will require a prospective, multi-center randomized study employing standardized treatment protocols. Risk stratification with longer follow-up durations will be required to determine the benefit of this technique. Still, we are encouraged with our preliminary data suggesting additions to DECRA may improve infection-free implant survival.

## 5. Conclusions

This study reports a higher success rate of DECRA with antibiotic-loaded CaSO_4_ beads compared to the current literature. Antibiotic beads provide advantage in selected groups that include A- or B-hosts and Limb score 1 or 2. In C-hosts, where the immune system is weak or Limb score 3, where the wound is compromised and leaks, locally placed antibiotic beads do not improve success. Most importantly, DECRA will unlikely be curative in cases of prior history of joint infection. In these difficult circumstances, one should consider an exchange protocol or continue with DECRA as a palliative procedure.

## Figures and Tables

**Table 1:** Factors included in multivariate logistic regression analysis for success of DECRA procedure.

Factor	*p*-Value
**Host-related factors**	
Age	0.986
Sex	0.697
BMI	0.769
Preoperative ESR	0.111
Preoperative CRP	0.272
McPherson Host Stage	**0.021**
**Limb-related**	
Type of procedure (Primary/Revision)	0.052
Type of joint infection (Post-operative/late hematogenous)	0.615
Intra-operative positivity of culture	0.574
McPherson Limb Score	**0.004**
Previous history of PJI	**0.000025**
**Time-related**	
Duration of symptoms	0.950
Time since index surgery	0.213

**Table 2:** Factors found to be significantly associated with success rate of DECRA procedure.

Host Factor	Success Rate
A-host	87.5% (14/16)
B-host	50% (11/22)
C-host	25% (1/4)
**Limb factor**	
Limb score 1	100% (8/8)
Limb score 2	58.6% (17/29)
Limb score 3	20% (1/5)
**Previous history of joint infection**	
Without previous history of joint infection	95% (19/20)
With previous history of joint infection	32% (7/22)

## Data Availability

The dataset used and analyzed during the current study are available from corresponding author on reasonable request.

## References

[1] Premkumar A., Kolin D.A., Farley K.X., Wilson J.M., McLawhorn A.S., Cross M.B., Sculco P.K. (2021). Projected Economic Burden of Periprosthetic Joint Infection of the Hip and Knee in the United States. J. Arthroplast..

[2] Kurtz S.M., Lau E., Watson H., Schmier J.K., Parvizi J. (2012). Economic burden of periprosthetic joint infection in the United States. J. Arthroplast..

[3] Petis S.M., Perry K.I., Mabry T.M., Hanssen A.D., Berry D.J., Abdel M.P. (2019). Two-Stage Exchange Protocol for Periprosthetic Joint Infection Following Total Knee Arthroplasty in 245 Knees without Prior Treatment for Infection. J. Bone Jt. Surg..

[4] Corona P.S., Vicente M., Carrera L., Rodríguez-Pardo D., Corró S. (2020). Current actual success rate of the two-stage exchange arthroplasty strategy in chronic hip and knee periprosthetic joint infection. Bone Jt. J..

[5] Stavrakis A., Mayer E.N., Devana S., Chowdhry M., Dipane M., McPherson E. (2022). Outcomes of Second-stage Reimplantation After Modular Knee Arthrodesis for Periprosthetic Joint Infection. JAAOS Glob. Res. Rev..

[6] Matar H.E., Bloch B.V., Snape S.E., James P.J. (2021). Outcomes of single- and two-stage revision total knee arthroplasty for chronic periprosthetic joint infection. Bone Jt. J..

[7] Stavrakis A.I., Mayer E.N., Devana S.K., Chowdhry M., Dipane M.V., McPherson E.J. (2022). Outcomes of Modular Knee Arthrodesis for Challenging Periprosthetic Joint Infections. Arthroplast. Today.

[8] McPherson E.J., Tontz W., Patzakis M., Woodsome C., Holtom P., Norris L., Shufelt C. (1999). Outcome of infected total knee utilizing a staging system for prosthetic joint infection. Am. J. Orthop..

[9] Hamad C., Chowdhry M., Sindeldecker D., Bernthal N.M., Stoodley P., McPherson E.J. (2022). Adaptive antimicrobial resistance, a description of microbial variants, and their relevance to periprosthetic joint infection. Bone Jt. J..

[10] McPherson E.J., Jennings J.A., Yunis O., Harris M.A., Dipane M.V., Curtin N.L., Chowdhry M., Wassef A.J., Bumgardner J.D., Noel S.P. (2022). Simulated large joint fluid model for evaluating intra-articular antibiotic delivery systems: Initial evaluation using antibiotic-loaded calcium sulfate beads. J. Bone Jt. Infect..

[11] McPherson E.J., Dipane M.V., Chowdhry M., Wassef A.J. (2021). Fabrication of antibiotic-loaded dissolvable calcium sulfate beads: An in vitro mixing lab utilizing various antibiotic mixing formulas. J. Bone Jt. Infect..

[12] McPherson E.J., Woodson C., Holtom P., Roidis N., Shufelt C., Patzakis M. (2002). Periprosthetic total hip infection: Outcomes using a staging system. Clin. Orthop. Relat. Res..

[13] Parvizi J., Gehrke T., Chen A.F. (2013). Proceedings of the International Consensus on Periprosthetic Joint Infection. Bone Jt. J..

[14] Prestinaci F., Pezzotti P., Pantosti A. (2015). Antimicrobial resistance: A global multifaceted phenomenon. Pathog. Glob. Health.

[15] Fisher C.R., Patel R. (2023). Profiling the Immune Response to Periprosthetic Joint Infection and Non-Infectious Arthroplasty Failure. Antibiotics.

[16] Sayan A., Kopiec A., Shahi A., Chowdhry M., Bullock M., Oliashirazi A. (2021). The Expanding Role of Biomarkers in Diagnosing Infection in Total Joint Arthroplasty: A Review of Current Literature. Arch. Bone Jt. Surg..

[17] Flaten D., Berrigan L., Spirkina A., Gin A. (2023). Risk of Treatment Failure for Prosthetic Joint Infections: Retrospective Chart Review in an Outpatient Parenteral Antimicrobial Therapy Program. Can. J. Hosp. Pharm..

[18] Le Vavasseur B., Zeller V. (2022). Antibiotic Therapy for Prosthetic Joint Infections: An Overview. Antibiotics.

[19] Longo U.G., Lalli A., Bandini B., Angeletti S., Lustig S., Budhiparama N.C. (2024). The influence of gut microbiome on periprosthetic joint infections: State-of-the art. J. ISAKOS.

[20] Chisari E., Cho J., Wouthuyzen-Bakker M., Parvizi J. (2022). Gut permeability may be associated with periprosthetic joint infection after total hip and knee arthroplasty. Sci. Rep..

[21] Ramirez J., Guarner F., Bustos Fernandez L., Maruy A., Sdepanian V.L., Cohen H. (2020). Antibiotics as Major Disruptors of Gut Microbiota. Front. Cell. Infect. Microbiol..

[22] Konstantinidis T., Tsigalou C., Karvelas A., Stavropoulou E., Voidarou C., Bezirtzoglou E. (2020). Effects of Antibiotics upon the Gut Microbiome: A Review of the Literature. Biomedicines.

[23] Francino M.P. (2016). Antibiotics and the Human Gut Microbiome: Dysbioses and Accumulation of Resistances. Front. Microbiol..

[24] Patangia D.V., Anthony Ryan C., Dempsey E., Paul Ross R., Stanton C. (2022). Impact of antibiotics on the human microbiome and consequences for host health. Microbiologyopen.

[25] Abdeen A., Della Valle C.J., Kendoff D., Chen A.F. (2022). The Paradox of Prosthetic Joint Infection and the Microbiome: Are Some Bacteria Actually Helpful?. Arthroplast. Today.

[26] Vaz K., Taylor A., Kendrick B., Alvand A. (2022). A guide to debridement, antibiotics, and implant retention. Ann. Jt..

[27] Tarity T.D., Xiang W., Jones C.W., Gkiatas I., Nocon A., Selemon N.A., Carli A., Sculco P.K. (2022). Do Antibiotic-Loaded Calcium Sulfate Beads Improve Outcomes After Debridement, Antibiotics, and Implant Retention? A Matched Cohort Study. Arthroplast. Today.

[28] McPherson E.J., Crawford B.M., Kenny S.G., Dipane M.V., Salarkia S., Stavrakis A.I., Chowdhry M. (2024). Point-of-Care Coating of Revision Femoral Stems with Antibiotic-Loaded Calcium Sulfate: Reduction in Infection After 2nd Stage Reimplantation but not with Aseptic Revisions. Arthroplast. Today.

[29] Hart C.M., Kelley B.V., Mamouei Z., Turkmani A., Ralston M., Arnold M., Bernthal N.M., Sassoon A.A. (2024). Antibiotic calcium sulphate beads lower the bacterial burden and prevent infection in a mouse model of periprosthetic joint infection. Bone Jt. J..

[30] Flierl M.A., Culp B.M., Okroj K.T., Springer B.D., Levine B.R., Della Valle C.J. (2017). Poor Outcomes of Irrigation and Debridement in Acute Periprosthetic Joint Infection with Antibiotic-Impregnated Calcium Sulfate Beads. J. Arthroplast..

[31] Silva M., Tharani R., Schmalzried T.P. (2002). Results of Direct Exchange or Debridement of the Infected Total Knee Arthroplasty. Clin. Orthop. Relat. Res..

[32] Calanna F., Chen F., Risitano S., Vorhies J.S., Franceschini M., Giori N.J., Indelli P.F. (2019). Debridement, antibiotic pearls, and retention of the implant (DAPRI): A modified technique for implant retention in total knee arthroplasty PJI treatment. J. Orthop. Surg..

[33] Thompson O., W-Dahl A., Stefánsdóttir A. (2022). Increased short- and long-term mortality amongst patients with early periprosthetic knee joint infection. BMC Musculoskelet. Disord..

[34] Okafor C.E., Nghiem S., Byrnes J. (2023). One-stage revision versus debridement, antibiotics, and implant retention (DAIR) for acute prosthetic knee infection: An exploratory cohort study. Arch. Orthop. Trauma Surg..

[35] McQuivey K.S., Bingham J., Chung A., Clarke H., Schwartz A., Pollock J.R., Beauchamp C., Spangehl M.J. (2021). The Double DAIR: A 2-Stage Debridement with Prosthesis-Retention Protocol for Acute Periprosthetic Joint Infections. JBJS Essent. Surg. Tech..

[36] Wignadasan W., Ibrahim M., Haddad F.S. (2023). One- or two-stage reimplantation for infected total knee prosthesis?. Orthop. Traumatol. Surg. Res..

[37] Chowdhry M., Dipane M.V., Duncan S.T., Pena D., Stavrakis A., McPherson E.J. (2024). Next generation sequencing identifies an increased diversity of microbes in post lavage specimens in infected TKA using a biofilm disrupting irrigant. Knee.

[38] Abosala A., Ali M. (2020). The Use of Calcium Sulphate beads in Periprosthetic Joint Infection, a systematic review. J. Bone Jt. Infect..

[39] De Meo D., Martini P., Pennarola M.F., Guarascio G., Capparuccia M.R., Iaiani G., Candela V., Gumina S., Villani C. (2023). Hydrogel Coating versus Calcium Sulphate Beads as a Local Antibiotic Carrier for Debridement Procedures in Acute Periprosthetic Joint Infection: A Preliminary Study. Gels.

[40] Caid M., Valk J., Danoff J. (2022). Irrigation Solutions in Total Joint Arthroplasty. Spartan Med Res. J..

[41] Springer B.D. (2022). Irrigation Solutions and Antibiotic Powders: Should I Use Them in Primary and Revision Total Joint Arthroplasty?. J. Arthroplast..

[42] Seta J.F., Pawlitz P.R., Aboona F., Weaver M.J., Bou-Akl T., Ren W., Markel D.C. (2024). Efficacy of Commercially Available Irrigation Solutions on Removal of *Staphylococcus aureus* and Biofilm from Porous Titanium Implants: An In Vitro Study. J. Arthroplast..

[43] Fröschen F.S., Randau T.M., Franz A., Molitor E., Hischebeth G.T.R. (2022). Microbiological Profiles of Patients with Periprosthetic Joint Infection of the Hip or Knee. Diagnostics.

[44] Weinstein E.J., Stephens-Shields A.J., Newcomb C.W., Silibovsky R., Nelson C.L., O’donnell J.A., Glaser L.J., Hsieh E., Hanberg J.S., Tate J.P. (2023). Incidence, Microbiological Studies, and Factors Associated with Prosthetic Joint Infection After Total Knee Arthroplasty. JAMA Netw. Open.

[45] de Lachica J.C.V., Reyes S.S.S., Ureña J.A.P., Fragoso M.A.R. (2022). Decrease in acute periprosthetic joint infections incidence with vancomycin-loaded calcium sulfate beads in patients with non-modifiable risk factors. A randomized clinical trial. J. ISAKOS.

[46] Maale G.E., Eager J.J., Mohammadi D.K., Calderon F.A. (2020). Elution Profiles of Synthetic CaSO_4_ Hemihydrate Beads Loaded with Vancomycin and Tobramycin. Eur. J. Drug Metab. Pharmacokinet.

[47] McPherson E., Dipane M., Sherif S. (2013). Dissolvable antibiotic beads in treatment of periprosthetic joint infection and revision arthroplasty-the use of synthetic pure calcium sulfate (Stimulan^®^) impregnated with vancomycin & tobramycin. Reconstr. Rev..

[48] Muthukrishnan G., Masters E.A., Daiss J.L., Schwarz E.M. (2019). Mechanisms of Immune Evasion and Bone Tissue Colonization That Make *Staphylococcus aureus* the Primary Pathogen in Osteomyelitis. Curr. Osteoporos. Rep..

[49] Schwarz E.M. (2022). What Are the Immune Responses That Allow Us to Live with Incurable Bone Infection, and How Can They Be Augmented to Improve Outcomes After Prosthetic Joint Infection?. J. Bone Miner. Res..

[50] Masters E.A., de Mesy Bentley K.L., Gill A.L., Hao S.P., Galloway C.A., Salminen A.T., Guy D.R., McGrath J.L., Awad H.A., Gill S.R. (2020). Identification of Penicillin Binding Protein 4 (PBP4) as a critical factor for *Staphylococcus aureus* bone invasion during osteomyelitis in mice. PLoS Pathog..

[51] Conway J., Delanois R.E., Mont M.A., Stavrakis A., McPherson E., Stolarski E., Incavo S., Oakes D., Salvagno R., Adams J.S. (2024). Phase 1 study of the pharmacokinetics and clinical proof-of-concept activity of a biofilm-disrupting human monoclonal antibody in patients with chronic prosthetic joint infection of the knee or hip. Antimicrob. Agents Chemother..

